# DOK7 Inhibits Cell Proliferation, Migration, and Invasion of Breast Cancer via the PI3K/PTEN/AKT Pathway

**DOI:** 10.1155/2021/4035257

**Published:** 2021-01-23

**Authors:** Changli Yue, Yuping Bai, Yingshi Piao, Honggang Liu

**Affiliations:** Department of Pathology, Beijing Tongren Hospital, Capital Medical University, Beijing Key Laboratory of Head and Neck Molecular Diagnostic Pathology, Beijing 100730, China

## Abstract

Recently, increasing attention has been paid to the correlation between the expression of downstream of kinase 7 (DOK7) and the occurrence and development of various tumors. In this study, we clarified the effects of DOK7 in breast cancer. First, we showed that DOK7 expression was obviously reduced in the breast cancer tissues and lower levels of DOK7 linked to more aggressive behaviors and worse prognosis of patients. Furthermore, DOK7 expression of various breast cancer cell lines was lower than that of human noncancerous MCF-10A cells. Overexpression of DOK7 inhibited proliferation, migration, and invasion, while silencing DOK7 expression promoted the malignancy of breast cancer. In addition, overexpression of DOK7 suppressed tumor proliferation and lung metastasis in animal models. Finally, to investigate the possible signaling mechanism, we first found that the level of p-AKT protein was extremely downregulated and the level of PTEN protein was remarkably upregulated after overexpressing DOK7 in breast cancer cells. Repression of PTEN expression using PTEN siRNA or SF1670 (PTEN inhibitor) rescued the tumor-inhibiting effect induced by DOK7 overexpression, suggesting that DOK7 inhibits proliferation, migration, and invasion of breast cancer cells though the PI3K/PTEN/AKT pathway. These results suggest that the downregulation of DOK7 may become a novel breast cancer therapeutic target.

## 1. Introduction

Breast cancer is the most frequently occurring malignancy and has a high mortality rate in cancer patients among worldwide [1]. Recently, many studies have illustrated that breast cancer is associated with various factors, including different lifestyles, virus infection, medical conditions, and oncogenic genes [2–7]. As well known, triple-negative breast cancer has great invasive ability, which makes it distant metastases [8, 9]. Despite tremendous progresses in breast cancer treatment, metastasis remains the most important causes of death in breast cancer patients [10]. Therefore, it has become an important research direction to find specific molecules that can be used as early diagnostic and prognosis markers and can be therapeutic targets.

The downstream of kinase (DOK) protein family, already has seven members, DOK1 to DOK7 [11–13], implicates in the intracellular signal transduction pathway of downstream of receptor tyrosine kinases (RTKs). DOK proteins share structural similarity of N-terminal contractile globulin homology (PH) and phosphotyrosine binding (PTB) domain and its C-terminal binds with SH2 target motifs [14, 15]. Recently, the correlation between DOK7 gene and tumors has been paid attention. DOK7 is closely related to malignant cancers, including lung cancer and glioma. A recent study has showed that the low level of DOK7 was responsible for poor prognosis in lung cancer patients and DOK7 played an important role on cell growth, migration, and invasion [13]. Another study also showed that silencing the expression of DOK7 significantly inhibited the development of glioma both in cells and in animal models [16]. However, the connection between the levels of DOK7 and the initiation of breast cancer has not been assessed yet.

In our recent findings, we displayed that the expression of DOK7 was remarkably reduced in clinical breast cancer tissues and the low levels of DOK7 were related to the bad clinical outcomes and prognosis in breast cancer patients. In addition, the overexpression of DOK7 inhibits proliferation, invasion, and migration of breast cancer. On the contrary, knocking down DOK7 expression promoted the malignancy of breast cancer. Mechanically, we confirmed that the protein levels of p-AKT were significantly reduced and the protein levels of PTEN were increased in DOK7 overexpression groups compared with the control groups. However, the repression of PTEN expression by PTEN siRNA or SF1670 (PTEN inhibitor) rescued the tumor-inhibiting effect induced by DOK7 overexpression. We demonstrated that DOK7 inhibits proliferation, migration, and invasion via the PI3K/PTEN/AKT pathway in breast cancer cells.

## 2. Materials and Methods

### 2.1. The Kaplan–Meier Plotter of Breast Cancer Patients

We used a Kaplan–Meier plotter (http://kmplot.com/analysis/) to analyze the relationship between DOK7 mRNA levels and prognosis in breast cancer patients. The database has the survival information from clinical cancer samples for over 54,000 genes across 21 cancer types, which include breast, lung, gastric, and ovarian cancers. The system has the survival information on 6,234 breast cancer patients, covering clinical factors such as ER status, PR status, HER2 status, intrinsic subtype, lymph node status, grade, TP53 status, pietenpol subtype, chemotherapy, and radiotherapy. In our study, we checked the association between DOK7 mRNA levels and prognosis (including overall survival (OS), relapse-free survival (RFS), and distant metastasis-free survival (DMFS)) in breast cancer samples using the Kaplan–Meier plotter analysis.

### 2.2. Cell Lines and Human Tissues

The human normal breast endothelial cell line MCF/10A and the breast cancer cell lines MCF-7, BT474, T47D, SKBR3, MDA-MB-231, HCC-1937, and SUM-1315 were obtained from the National Infrastructure of Cell Line Resource (Beijing, China). The culture medium for MCF/10A cells is Endothelial Cell Medium (ScienCell, USA). MCF-7, MDA-MB-231, T47D, and SUM-1315 cells were cultured in the DMEM medium containing 10% fetal bovine serum (Hyclone) and 1% penicillin and streptomycin (Beyotime Biotechnology, China) at 37°C with 5% CO_2_. BT474, SKBR3, and HCC-1937 cells were cultured in the RPMI-1640 medium containing 10% fetal bovine serum (Hyclone) and 1% penicillin and streptomycin (Beyotime Biotechnology, China) at 37°C with 5% CO_2_. 68 breast cancers and matched paracancer tissues were collected from March 2016 to January 2019 of the Department of Pathology of Beijing Tongren Hospital. All the specimens were approved by the Ethics Committee of Beijing Tongren Hospital, and informed consent was provided from every patient.

### 2.3. Cell Proliferation

CCK8 assay (Dojindo, Japan) was used to analyze cell proliferation. 1 × 10^4^ cells were cultured in 96-well plates for 24 hours, 48 hours, 72 hours, and 96 hours. Then, after incubation, 10 *μ*L of CCK8 reagent was added into every well of 96-well plates and incubated for 1 hour. After incubation of CCK8 reagent, we measured the OD value at 450 nm.

### 2.4. Construction of Stable Cell Lines

MDA-MB-231 and SKBR3 stable cell lines were transfected with pcDNA3.1-DOK7 or pcDNA3.1 plasmids and selected with puromycin for 1 month. The plasmids were obtained from OBiO Technology Co. Ltd. (Shanghai, China). Specifically, cells (70% confluence) were seeded into culture flasks, and plasmid transfection was carried out using Lipofectamine 2000 (Invitrogen) for 48–72 hours. Then, these transfected cells were selected with 5 *μ*g/mL puromycin for 1 month.

MCF-7 and SKBR3 cell lines stably silencing DOK7 were constructed using a lentiviral shRNA technique; shRNAs (DOK7 shRNA or scrambled shRNA) were obtained from GenePharma Company (Shanghai, China). Specifically, 293T cells (30%–50% confluence) were seeded in 6-well plates and transfected with DOK7 shRNA or scrambled shRNA for 24 hours using Lipofectamine 2000 (Invitrogen). Then the supernatant from transfected 293T cells was collected and filtered and added to MCF-7 and SKBR3 cells (50% confluence) for 48 hours, followed by puromycin (5 *μ*g/ml) selection for one month.

### 2.5. Transwell Assays

Transwell assays were used to analyze the migration and invasion of cancer cells. For migration assay, 1 × 10^5^ breast cancer cells were seeded on the upper 24-well transwell chambers (Millipore) without Matrigel gel (BD Biosciences) and cultured for 24 hours. For invasion assay, (2–5) × 10^5^ breast cancer cells were cultured on the upper 24-well chambers with Matrigel gel (BD Biosciences) and incubated for 48 hours. After incubation, the cells moved to the bottom of the 24-well chamber, followed by fixing with 4% formaldehyde and dyeing with crystal violet reagent.

### 2.6. Quantitative Real-Time PCR (qRT-PCR)

Total RNA was extracted using the Qiagen RNeasy Mini kit. Total RNA (1 *µ*g) was reverse-transcribed using the Reverse Transcription System (Takara). qRT-PCR was performed with the SYBR Green kit (Takara). Primers for PCR DOK7: 5′-TGCCAAGCGGATTCATCTTTG-3′ (forward), 5′-GACGATGCAGTCGAACAGGAA-3′ (reverse); and primers for GAPDH: 5′-TGACTTCAACAGCGACACCCA-3′ (forward), 5′-ACCCTGTTGCTGTAGCCAAA-3 (reverse).

### 2.7. Western Blotting Assay

Protein expression was examined using western blotting assay. The proteins were collected using protein digestion buffer (strong RIPA buffer (Beyotime Biotechnology, China) with protease inhibition (ThermoFisher, USA)). Then, protein concentration was evaluated using a BSA kit (ThermoFisher, USA). 30 *μ*g protein samples were added into each lane, followed by separating in gels and transferring to PVDF membranes (Millipore). Then, membranes were blocked with 5% milk and incubated with primary antibodies (diluted with 5% BSA) (anti-DOK7, Abcam, ab75049, 1 : 1000 dilution; anti-AKT, Cell Signaling Technology, #4685, 1 : 1000 dilution; anti-p-AKT, Cell Signaling Technology, #4060, 1 : 1000 dilution; anti-PTEN, Cell Signaling Technology, #9188, 1 : 1000 dilution; and anti-*β*-Actin, Bioworld, BS6007MH, 1 : 5000 dilution) overnight at 4°C. The next day, incubation of secondary antibodies (diluted with 3% milk) (Cell Signaling Technology, 1 : 5000 dilution) was performed at room temperature for 2 hours.

### 2.8. Xenograft Tumor Models

Four-week-old female nude mice were obtained from Beijing Vital River Laboratory Animal Technology Co., Ltd. For primary tumors, MDA-MB-231 stably overexpressing DOK7 or vector control cells (5 × 10^6^ cells in 50 *µ*L DMEM medium) were inoculated into mammary fad pads (MFP) of 6 nude mice, respectively. The formula of tumor volume is as follows: [(length) × (width) × (length + width/2) × 0.526 = volume]. The sizes of tumors and the body weights of mice were recorded every three days. After 30 days, tumors were excised and weighed. For the metastasis model, DOK7-overexpressed or controlled stable MDA-MB-231 cells (2 × 10^5^ cells in 50 *µ*L PBS) were injected into the tail vein of 5 nude mice, respectively. After 45 days, all lungs of mice were collected. For the PTEN inhibitor model, controlled stable MDA-MB-231 cells (5 × 10^6^ cells in 50 *µ*L DMEM medium) were inoculated into mammary fad pads (MFP) of 4 nude mice and DOK7-overexpressed stable MDA-MB-231 cells (5 × 10^6^ cells in 50 *µ*L DMEM medium) were inoculated into mammary fad pads (MFP) of 8 nude mice. When the tumors turned palpable, the mice were randomized into three groups: (i) Vector/DOMSO group, intraperitoneal injection of DMSO (500 *µ*L) daily in 4 mice; (ii) DOK7/DMSO group, intraperitoneal injection of DMSO (500 *µ*L) daily in 4 mice; and (iii) DOK7/SF1670 group, intraperitoneal injection of SF1670 (10 *μ*mol/kg diluted in 500 *µ*L DMSO) daily in 4 mice [17]. After 21 days, tumors were excised and weighed. The animal experiments were performed following the Animal Care Committee of Beijing Tongren Hospital.

### 2.9. Immunohistochemistry

The tissues were fixed with 4% formaldehyde for 24 hours and embedded with paraffin, and then immunohistochemistry was performed. Specifically, slides were boiled in antigen unmasking solution (pH 6.0) for 20 min and blocked with 0.3% H_2_O_2_ for 20 min. Then, the slides were incubated with 5% BSA and followed with incubation of primary antibodies (anti-Ki-67 (Abcam), ab15580, 1:200 dilution; anti-PCNA (Proliferating Cell Nuclear Antigen, Dako), M0879, 1 : 50 dilution) overnight at 4°C. The next day, incubation of secondary antibodies was performed at 37°C for 1 hour, followed by treating with DAB chromogen.

### 2.10. Statistical Analysis

GraphPad Prism 7.0 was used to make all graphs and statistical analyses. The two-tailed Student's *t*-test and Log-rank test were used to perform statistical analysis. *P* value <0.05 was judge to indicate statistical significance.

## 3. Results

### 3.1. DOK7 Is Significantly Downregulated in Breast Cancer Tissues and Is Associated with Clinical Outcomes

**Figure 1:**
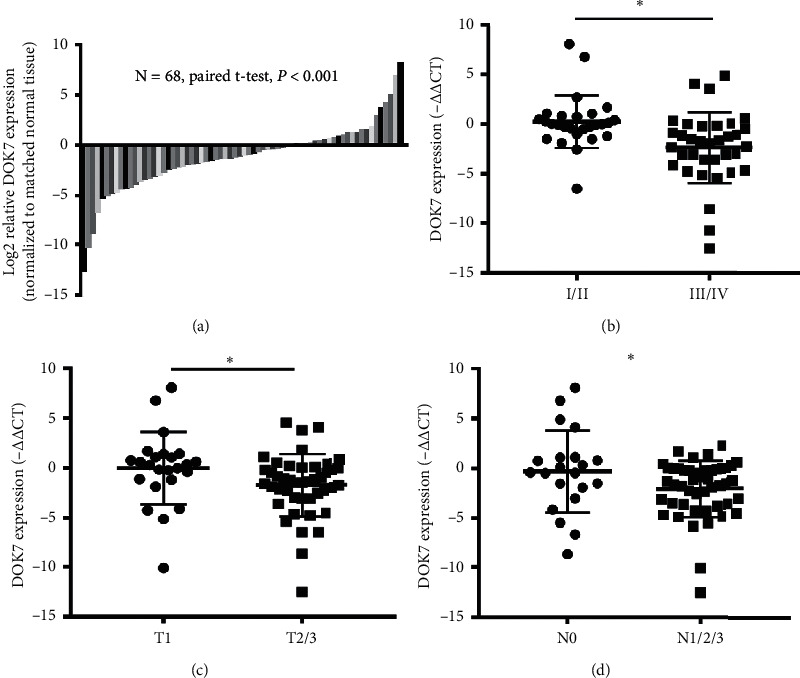
DOK7 is significantly downregulated in breast cancer tissues and is associated with clinical outcomes. (a) The mRNA levels of DOK7 in 68 paired normal and breast cancer tissues were evaluated using qRT-PCR assay. The mRNA levels of DOK7 were shown as log_2_ fold-change vs adjacent tissues. (b) Expression of DOK7 in 68 paired normal and breast cancer tissues with different TNM stages (I/II stage or III/V stage) was evaluated using qRT-PCR assay. (c) The mRNA levels of DOK7 in 68 paired normal and breast cancer tissues with different T stage statuses (T1 or T2/3) were measured using qRT-PCR assay. (d) The mRNA levels of DOK7 in 68 paired normal and breast cancer tissues with different lymph node metastases (N0 or N1/2/3) were checked using qRT-PCR assay. ^*∗*^*P* < 0.05; ^*∗∗*^*P* < 0.01.

To inquire the relationship between the levels of DOK7 and the clinical features of breast cancer patients, we compared the mRNA levels of DOK7 in 68 paired normal and breast cancer tissues. In matched breast tissue pairs obtained from 68 breast cancers (Table 1), DOK7 expression was obviously decreased in tumors (Figure 1(a)). To investigate whether the level of DOK7 is related to the aggressive behavior in breast cancer patients, we analyzed the association between DOK7 levels and different clinical outcomes (TNM stage, tumor diameter, and lymph node metastasis). The results showed that the breast cancer tissues of the higher stage (III/IV stage) expressed significantly lower levels of DOK7 than those of I/II stage subgroups (Figure 1(b)). Similarly, DOK7 mRNA expression was significantly reduced in large tumors (T1 stage, tumor diameter >2 cm) compared with tumors with the diameter ≤2 cm (T2/3 stage, Figure 1(c)). Furthermore, patients with the high lymph node metastasis (N1/2/3) group had lower expression of DOK7 than that of the low lymph node metastasis (N0) (Figure 1(d)). These data indicate that lower DOK7 expression is related to more aggressive behaviors in breast cancer patients.

### 3.2. Low DOK7 Expression Leads to Poor Prognosis of Breast Cancer Patients

**Figure 2:**
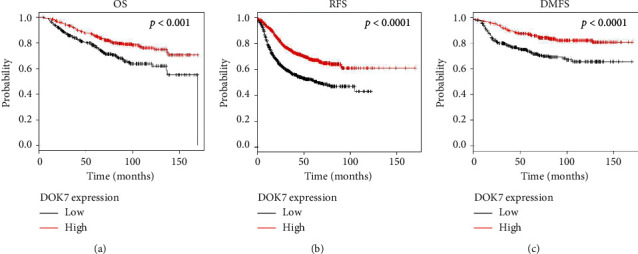
Low DOK7 expression leads to poor prognosis of breast cancer patients. The relationship between the expression of DOK7 and overall survival (OS) (a), relapse-free survival (RFS) (b), and distant metastasis-free survival (DMFS) (c) was performed using a Kaplan-Meier plotter (KMplot, http://kmplot.com/analysis/).

To further confirm the effects of DOK7 expression on clinical prognosis in breast cancer patients, we also viewed the relationship between the levels of DOK7 and survival of breast cancer patients by a Kaplan-Meier plotter (KMplot, http://kmplot.com/analysis/). Patients with lower levels of DOK7 had worse overall survival (OS) (*P* < 0.001) in 626 breast cancer patients (314 samples in low levels of DOK7 and 312 samples in high levels of DOK7) (Figure 2(a)). In addition, similar results were obtained using relapse-free survival (RFS) (*P* < 0.0001) in 1764 breast cancer patients (886 samples in low levels of DOK7 and 878 samples in high levels of DOK7) (Figure 2(b)). Similarly, higher levels of DOK7 had better distant metastasis-free survival (DMFS) than those with low DOK7 expression (*P* < 0.0001) in 664 breast cancer patients (333 samples in low levels of DOK7 and 331 samples in high levels of DOK7) (Figure 2(c)). All these data suggested that low levels of DOK7 resulted in a bad prognosis in breast cancer patients.

### 3.3. Overexpression of DOK7 Inhibits Proliferation, Migration, and Invasion of Breast Cancer Cells

**Figure 3:**
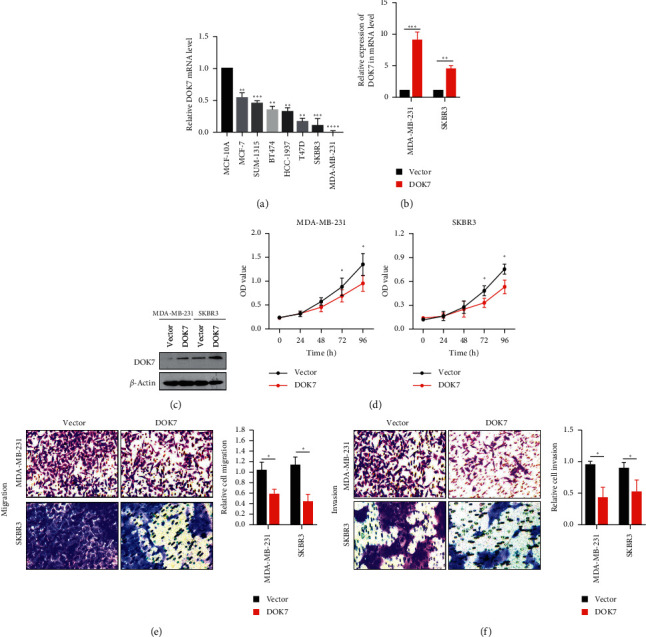
Overexpression of DOK7 inhibits proliferation, migration, and invasion in breast cancer cells. (a) The levels of DOK7 in a human noncancerous cell line (MCF-10A) and various breast cancer cell lines (MCF-7, SUM-1315, BT474, HCC-1937, T47D, SKBR3, MDA-MB-231) were analyzed using qRT-PCR assay. The expressions of DOK7 levels were normalized to MCF-10A normal mammary epithelial cells. (b) MDA-MB-231 and SKBR3 cells were transfected with DOK7-overexpressed or controlled plasmids, and the mRNA levels of DOK7 were evaluated using qRT-PCR assay. (c) The protein levels of DOK7 were analyzed using western blotting assay. (d) Cell viability in vector groups and DOK7 overexpression groups was analyzed using CCK8 assay. (e) Migration assay in the vector group and DOK7 group was performed using transwell assay without Matrigel gel. (f) Invasion assay in the vector group and DOK7 group was performed using transwell assay with Matrigel gel. ^*∗*^*P* < 0.05; ^*∗∗*^*P* < 0.01; ^*∗∗∗*^*P* < 0.001; and ^*∗∗∗∗*^*P* < 0.0001.

After we identified the relationship between the levels of DOK7 and clinical outcomes and prognosis in breast cancer patients, we inquired the mRNA levels of DOK7 in various breast cancer cell lines (MCF-7, SUM-1315, BT474, HCC-1937, T47D, SKBR3, MDA-MB-231) and a human noncancerous cell line (MCF-10A). The results showed that the mRNA levels of DOK7 of breast cancer cells were much lower than MCF-10A cells by qRT-PCR assay (Figure 3(a)). MDA-MB-231 and SKBR3 cells had lowest DOK7 expression among all breast cancer cells, so we overexpressed DOK7 in these two cell lines. After transfection and selection, the transfection efficiency showed that the expression of DOK7 in overexpression groups of MDA-MB-231 and SKBR3 cells both in mRNA and protein levels was significantly evaluated compared with vector groups (Figure 3(b) and 3(c)). Furthermore, we found that cellular growth in MDA-MB-231 and SKBR3 cells was repressed by DOK7 overexpression by CCK8 assay (Figure 3(d)). The transwell assay results showed that the overexpression of DOK7 inhibited migration and invasion both in MDA-MB-231 and SKBR3 cells (Figure 3(e) and 3(f)).

### 3.4. Knocking Down DOK7 Promotes Proliferation, Migration, and Invasion of Breast Cancer Cells

**Figure 4:**
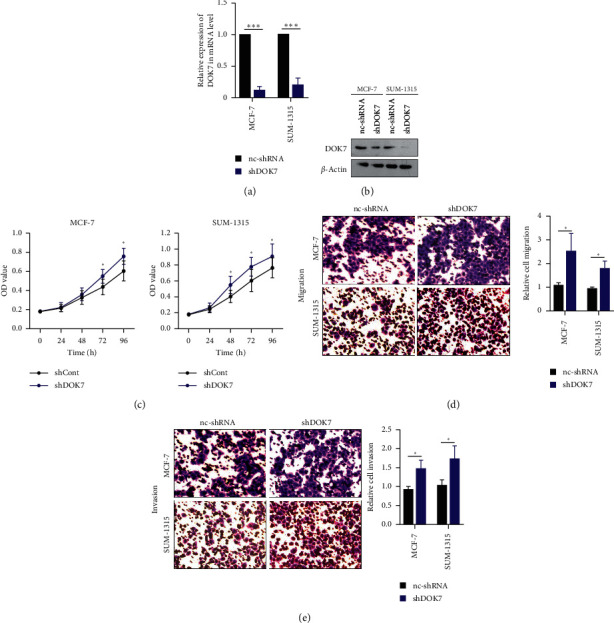
Knocking down DOK7 promotes proliferation, migration, and invasion in breast cancer cells. (a) MCF-7 and SUM-1315 cells were transfected with DOK7 shRNA or scrambled control-shRNA and the qRT-PCR assay was used to measure the mRNA levels of DOK7. (b) Western blotting assay was used to check the protein levels of DOK7. (c) Cell viability in the nc-shRNA group (cells transfected with scrambled control-shRNA) and shDOK7 group (cells transfected with DOK7 shRNA) was analyzed using CCK8 assay. (d) Migration assay in the nc-shRNA group and shDOK7 group was performed using transwell assay without Matrigel gel. (e) Invasion assay in the nc-shRNA group and shDOK7 group was performed using transwell assay with Matrigel gel. ^*∗*^*P* < 0.05; ^*∗∗∗*^*P* < 0.001.

From above results, MCF-7 and SUM-1315 cells had the highest DOK7 expression among all breast cancer cells; we deleted DOK7 expression by transfecting with shRNAs in these two cell lines. Silencing DOK7 expression in MCF7 and SUM-1315 cells significantly reduced DOK7 expression was found using qRT-PCR and western blotting assays (Figure 4(a) and 4(b)). In addition, DOK7 depletion significantly promoted the cell growth of MCF7 and SUM-1315 cells compared with the control groups was found using CCK8 assay (Figure 4(c)), suggesting that silencing DOK7 expression promoted cellular growth of breast cancer cells *in vitro*. Furthermore, MCF7 and SUM-1315 cells with DOK7 shRNAs exhibited higher migration and invasion ability than control cells (Figure 4(d) and 4(e)). These data showed that knocking down the DOK7 expression of breast cancer cells resulted in promoting proliferation, migration, and invasion.

### 3.5. DOK7 Inhibits Tumor Growth and Metastasis of Breast Cancer *In Vivo*

**Figure 5:**
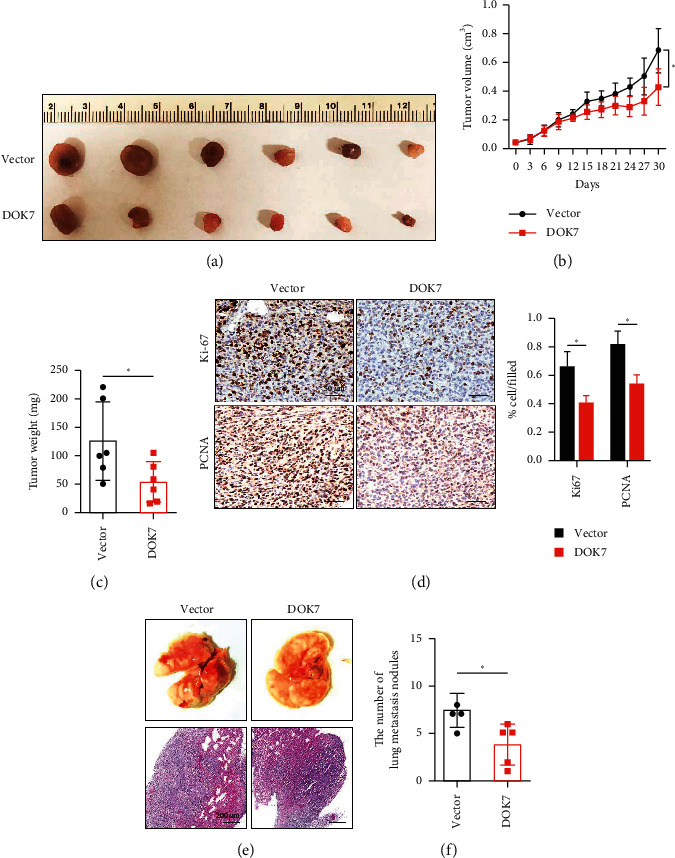
DOK7 inhibits tumor growth and metastasis of breast cancer in vivo. (a) DOK7-overexpressed and controlled MDA-MB-231 cells were injected into the mammary fad pads of nude mice. The tumor sizes (b) and tumor weights (c) in the control group and DOK7 overexpression group were calculated. (d) The expression of Ki-67 and PCNA in the control group and DOK7 overexpression group was evaluated using immunohistochemistry assay. (e) DOK7-overexpressed and controlled MDA-MB-231 cells were injected into the tail vein. (f) The number of lung metastatic nodules of mice was calculated. Scale bar = 50 *µ*m in (d). ^*∗*^*P* < 0.05; ns, no significant.

We have confirmed that knockdown of DOK7 inhibited the cell proliferation and cell invasion and migration abilities *in vitro* (Figure 3). Next, to further verify the effects of DOK7 overexpression on tumorigenic property *in vivo*, the mammary fad pads (MFP) of 12 nude mice were inoculated with DOK7-overexpressed or controlled MDA-MB-231 cells. The growth of tumors was obviously slower in the DOK7 overexpression group than in the vector group (Figure 5(a)). The tumor sizes and tumor weights were significantly smaller in the DOK7 overexpression group compared with the control group (Figure 5(b) and 5(c)). Moreover, immunohistochemistry assay showed that DOK7-overexpressed MDA-MB-231 cells had lower protein levels of Ki-67 and PCNA, which were the common proliferation-related markers (Figure 5(d)), indicating DOK7 overexpression suppressed cell proliferation *in vivo*. We next tested metastasis behavior changes using tail vein injection of MDA-MB-231 cells. There is a dramatic decrease in the quantification of pulmonary metastasis nodules in the DOK7 overexpressed group (Figure 5(e) and 5(f)). Taken together, these data presented that the elevated expression of DOK7 is crucial for inhibiting tumor progression and metastasis of breast cancer.

### 3.6. DOK7 Represses Proliferation, Migration, and Invasion of Breast Cancer via the PI3K/PTEN/AKT Pathway

**Figure 6:**
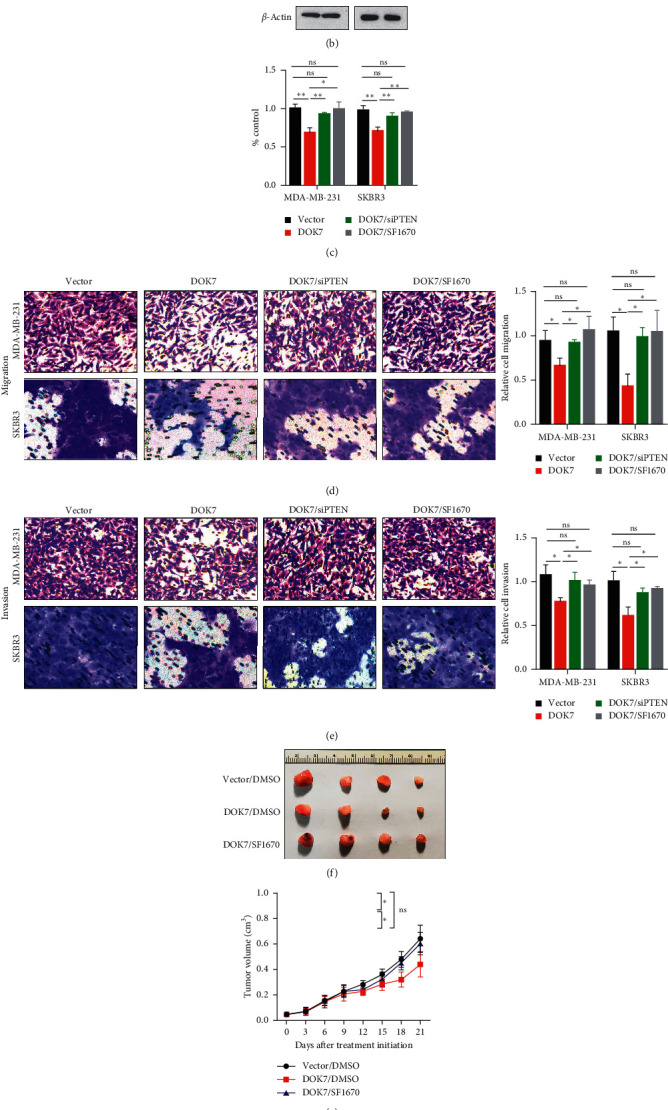
DOK7 inhibits proliferation, migration, and invasion of breast cancer via the PI3K/PTEN/AKT pathway. (a) Western blotting assay was performed on PI3K/PTEN/AKT pathway-related proteins (AKT, p-AKT, PTEN). (b) Knocking down PTEN expression by siRNAs in MDA-MB-231 and SKBR3 cells was detected using western blotting assay. (c) Cell viability in the vector group, DOK7 group, DOK7/siPTEN group (overexpression DOK7 cells were transfected with PTEN siRNA), and DOK7/SF1670 group (overexpression DOK7 cells were treated with 10 *µ*M SF1670 for 24 h) were analyzed using CCK8 assay. Cells in vector group, DOK7 group, DOK7/siPTEN group, and DOK7/SF1670 group were performed using (d) migration and (e) invasion assays. (f)–(h) The tumor sizes and tumor weights in vector, DOK7, and DOK7/SF1670 groups were calculated. ^*∗*^*P* < 0.05; ^*∗∗*^*P* < 0.01; ns, no significant.

To investigate the molecular mechanisms how DOK7 expression mediated tumor growth and metastasis, we checked PI3K/PTEN/AKT pathway-associated proteins in DOK7-overexpressed or controlled MDA-MB-231 and SKBR3 cells. Experimental results discovered that the protein level of p-AKT was extremely downregulated and the protein level of PTEN was significantly upregulated in DOK7 overexpression groups compared with the vector control groups (Figure 6(a)). We then investigated whether the PI3K/PTEN/AKT pathway was responsible for the DOK7-induced suppression of cell viability, migration, and invasion. To do this, we first knocked down PTEN expression by siRNA methods in MDA-MB-231 and SKBR3 cells (Figure 6(b)). Interestingly, overexpression of DOK7 restrained cell proliferation both in MDA-MB-231 and SKBR3 cells, while repression of PTEN expression using PTEN siRNA or SF1670 (PTEN inhibitor) rescued the tumor-inhibiting effect induced by DOK7 overexpression (Figure 6(c)). Furthermore, we also performed transwell assay and found that suppression of PTEN expression by PTEN siRNA or SF1670 could also reversed DOK7 overexpression, mediating migration and invasion inhibition (Figure 6(d) and 6(e)). These data suggested that DOK7 inhibits proliferation, migration, and invasion of breast cancer cells via the PI3K/PTEN/AKT pathway. Finally, to further confirm whether DOK7 could repress tumor growth of breast cancer *in vivo*, we injected MDA-MB-231 cells into the mammary fad pads of nude mice and treated with DMSO or SF1670 (10 *μ*mol/kg) [18]. The results showed that tumor volume and weights decreased in the DOK7 overexpression group compared with the control vector group, while inhibiting PTEN expression using the PTEN inhibitor SF1670 rescued DOK7 overexpression-induced tumor growth (Figure 6(f)–6(h)).

## 4. Discussion

The downstream of tyrosine kinase (DOK) family is composed of seven members, DOK1 to DOK7, and some of them have the effects on the negative regulation of tumor signaling pathways [15, 19–21]. Recently, several studies of other DOK proteins such as DOK1, DOK2, and DOK6 participate in the occurrence and development of breast cancer. In breast cancer samples, the mRNA levels of DOK1 were significantly decreased in tumors compared with adjacent normal tissues and DOK1 expression was correlated between DOK1 expression and c-cerbB-2 status [22]. The negative expression of DOK2 was related to poor prognosis and bad clinical parameters of 285 patients with breast cancer, suggesting that DOK2 can be an independent prognostic factor in breast cancer [23]. Tamara et al. showed that DOK6 acts as a tumor suppressor in human breast cancer [24]. There was also a significant reduction of DOK3 in lung cancer and aggressive histiocytic sarcoma [25, 26]. The other subgroup of the DOK family, consisting of DOK4 and DOK5, is mainly expressed in the nervous system [27, 28].

Recently, increasing attention has been paid to the association with DOK7 and the progression of cancers. Previous studies demonstrated that DOK7 was downregulated in a variety of cancer tissues and low levels of DOK7 closely linked to poor prognosis in lung cancer patients [9, 29]. Zhang et al. also discovered that high levels of DOK7 in patients with acute myeloid leukemia (AML) had longer OS than the low DOK7 expressers [18]. A recent study showed that DNA hypermethylation of DOK7 had been detected in breast cancer samples (blood from breast cancer patients, breast cancer tumors, and breast cancer cell lines), suggesting that DOK7 promoter methylation could become a novel molecular biomarker in early detection of breast cancer [18, 25].

Growing evidences indicate that DOK7 plays a vital role on breast tumorigenesis. Here, for the first time, we exhibited that DOK7 expression reduced in breast cancer tissues and lower levels of DOK7 were related to more aggressive clinical behaviors and worse prognosis of breast cancer. Here, we overexpressed DOK7 expression and found that DOK7 inhibited proliferation and metastasis both *in vitro* and *in viv*o. On the contrary, knocking down the expression of DOK7 promoted the malignancy in breast cancer cells *in vitro*.

At present, there are several mechanisms of weak expression of DOK7 in tumors. DNA hypermethylation of DOK7 had been detected in breast cancer samples, including blood, tumor tissues, and cultured cells, and it is considered to be one of the downregulation mechanisms in DOK7 expression [16]. Furthermore, DNMT1, one of the major members from the DNA methyltransferase (DNMT) family, is a DNA methyltransferase and DOK7 methylation was enhanced by increasing DNMT1 expression, which resulted in the downregulation of DOK7 expression [25]. A recent study suggested that the expression of DOK7 proteins was associated with AKT and ERK pathways [29]. However, the specific molecular mechanisms are still poorly defined.

PI3K/AKT/PTEN is an important pathway on the initiation and development in various tumors, which plays an important role in promoting tumor growth and suppressing apoptosis of tumor cells [30, 31]. PTEN, a famous tumor inhibitor, is one of the critical members of PI3K/AKT/PTEN pathways. To investigate whether the PI3K/AKT/PTEN pathway participated in DOK7-induced cell growth, invasion, and migration in breast cancer cells, we confirmed that the protein level of p-AKT was extremely lower and the protein level of PTEN was obviously higher in DOK7 overexpression groups than the vector control groups in breast cancer cells. Repression of PTEN expression by PTEN siRNAs or SF1670 (PTEN inhibitor) rescued the tumor-inhibiting effect of DOK7 overexpression, suggesting that DOK7 inhibits proliferation, migration, and invasion of breast cancer cells though the PI3K/PTEN/AKT pathway.

In conclusion, this study displayed that DOK7 was lowly expressed in breast cancer tissues, suggesting that DOK7 was a potential tumor-suppressor gene. Specifically, we confirmed that the low levels of DOK7 were related to bad clinical outcomes and poor prognosis of patients with breast cancer; therefore, DOK7 had a potential value as a molecular biomarker in prognosis prediction of breast cancer. Mechanistic studies showed that DOK7 inhibits proliferation, migration, and invasion of breast cancer cells via PI3K/PTEN/AKT pathway. These results suggested that the downregulation of DOK7 may become a novel breast cancer therapeutic target.

## Figures and Tables

**Table 1:** The clinicopathological features of breast cancer patients.

Parameters	Total
Age (years)	
<50	23
>50	45

TNM stage	
I	14
II	13
III	36
IV	5

Tumor diameter	
T1	40
T2	19
T3	9

Lymph node metastasis	
N0	21
N1/2/3	47

Distant metastasis	
M0	63
M1	5

## Data Availability

The data used to support the findings of this study are included within the article.
